# The Thermometer in Disease

**Published:** 1872-09

**Authors:** F. M. Hamlin

**Affiliations:** Union Springs, N. Y.


BUFFALO

### Medical and Surgical Journal.

VOL XII. SEPTEMBER, 1872. No. 2

### Original Communications.

—:o:—

ART. I.—*The Thermometer in Disease.* By F. M. HAMLIN, M. D. Union Springs, N. Y.

Read before the Central New York Medical Association.

Any instrument or appliance of modern art or science which may be used in Medicine to solve mixed questions of cause and effect, to remove from the domain of the *probable* to the *positive*, thereby aiding in rendering Medicine an exact science, ought to be hailed by the profession with delight.

The rendering of symptoms *objective* which were *subjective* has been followed by rapid strides in modern Medicine. With how much more certainty we deal with the diseases of the chest and abdomen since the application of the principles of acoustics; or of the eye, ear and throat since we use instruments divised according to the laws of optics.

But no instrument, in my opinion, is so generally or widely useful as the thermometer. A very large proportion of the diseases a physician is called upon to treat, are accompanied by fever or increased heat of the body. Now the measure of this degree of heat is the measure of the difference between the existing condition and that of health.

From time without date the hand has been used to detect these changes of temperature; but from the nature of the case it is a faulty and unreliable guide, for so much depends upon existing circumstances. If the hand be cold, or damp, or hardened, the sensations are very different when applied to the same substance. Then again the sensation imparted at the time of one observation can not be remembered exactly for comparison with that of another; and again the temperature of exposed parts of the body as the hands, face, &c., does not indicate, from unequal circulation, the actual degree of heat of the body. With such conditions the result of hand-thermometry, of a necessity, must be fallacious and unsatisfactory. The thermometer at once overcomes these obstacles and gives the true amount of heat.

The use of this instrument may be regarded in two aspects. 1st. As an aid in the diagnosis and prognosis of disease.

2d. By repeated and continued use the normal character of most febrile affections may be ascertained, thus contributing an important part to the natural history of disease.

Before considering temperature in disease it will be necessary to know the normal heat of the body and its variations in health. The average is 98.4° with slight deviations between morning and evening, and it may range from 97.82° to 99° without morbid action being present.

In tropical regions it is about one degree higher than in temperate. In the tropics it is lowest mornings and highest during the day; in the temperate it is highest in the morning on rising and lowest at midnight. Active exercise and increased heat of the atmosphere cause a slight rise, prolonged exposure to cold, and long continued mental exertion lessen the heat; and a full meal or dose of alcohol will at first cause a fall in the temperature, but it rises again as digestion and absorption progress.

There is a certain degree of relation between the increase of heat and the frequency of the pulse. An increase of one degree will be followed by an increase of ten pulsations, that is at 98° the pulse is 70, at 99° it is 80, at 100° it is 90, and so on. But this will be so varied from, that little dependence can be placed upon it; thus, in a case of erysipelas of the face under my care the temperature at one time was up to 106¼°, but the pulse was 122; also in a case of pneumonia with a fever heat of 104½° the pulse was 94. But in these cases the pulse may have been modified by the medicines exhibited.

Could a positive law of relation be discovered, it would open a wide field for investigation in regard to the effect of medical agents upon the circulation. If the temperature should remain uninfluenced, but the pulse reduced by the administration of a remedy, I should regard it as doing a good work, especially if it be of a tonic and sustaining nature; and better still if by it both fever and pulse are reduced. In stages of callapse and dissolution there will be a wide departure. It will be seen by these facts that no general law can be established, as yet at least, but it will be well to observe clearly the degree of correspondence in each individual case.

The observation should be made by placing the bulb of the thermometer in the axilla, making sure that the skin and it are in direct contact, where it should remain from three to five minutes, the time depending upon the instrument used. Care should be taken that the thermometer is a reliable one. The best are those which are self-registering, as no examination need be made while in place, and covered by the clothing. Those which are not must be noted before removal from the person. An excellent instrument may be obtained of Tiemann & Co., New York.

In acute cases two observations should be made daily, morning and evening, and at the same hour as nearly as possible; in chronic diseases once a day will be sufficient. A continued series of observations will sometimes be necessary to settle or clear up the diagnosis of some diseases. But oftimes one will be of great aid, thus, a temperature of 99° simply shows the individual is unwell; when above 101° to 105° the febrile symptoms are severe; above 105° the patient is in imminent danger; above 106° to 108° or 109° a fatal issue may be expected.

An individual yesterday well but who to day has a temperature of 104° is either suffering from an attack of ephemeral, or malarious fever, or an acute inflammation of some internal organ, its precise location, other signs must determine. Should it rise to 106° the first day, it certainly is an ague or some form of malarious fever. If after the eruption of measles has faded, there is still a high temperature; it may be determined that some complication exists. A rise in the declining stage of scarlatina shows the supervention of some of the sequelæ, as nephretis.

In a case of pneumonia if the temperature has begun to decline and then rises again it shows the invasion of another lobe. In this disease if the heat does not rise above 104° it is a mild attack; if it should rise and continue above 104° any length of time, with a quick pulse it is not devoid of danger. In a case presenting the physical signs of pneumonia, if the temperature does not rise above 101.7° it may be concluded no soft infiltrating exudation has taken place. In acute rheumatism a rise to 104° indicates danger of cardiac inflammation.

In nearly all of the febrile affections there is an decrease from night to morning, and an increase from morning to night, this is a favorable symptom even though febrile action is severe; but where the morning temperature is as high or higher than the evening previous the prognosis must be guarded, for it is a bad omen.

A temperature of 103° to 104° in any disease shows that its course is not checked. Convalescence can not be declared established, until the morning and evening temperature remain at the normal degree, that is, it can not be said to exist until the morbid process ceases. This will be indicated most quickly and positively by the thermometer.

Diseased action may terminate slowly, lysis, or rapidly, crisis. If it be slow and regular it is a sure sign of convalescence soon to be established; but should it be slow and irregular constant care and watchfulness should be used to prevent complications. It should be known that in diseases terminating by crisis the temperature may fall *below* the normal point, as to 95°. Such stages of collapse should be treated by stimulants and warm applications, the thermometer thus giving an important clue to treatment. A sudden fall in a case of typhoid fever below 98° is indicative of intestinal hemmorrhage. A quick fall to, or near the normal degree in typhoid fever, pneumonia, or measles, points to the near termination of the disease.

A fall of temperature below the healthy degree is rare. It sometimes occurs in the morning remissions of remittents, in the apyrexia of intermittents, in stages of collapse, in chronic wasting diseases, as cancer, and sometimes on the approach of death. In the algide stage of cholera the temperature varies in different parts of the body. Dr. Sutton states, that the axilla may be at 92° and in the rectum 102°. The axilla at 95° or 96° shows imperfect reaction taking place. A range of 14¼° has been noted in this disease, the extremes being 91.2° and 105.6°.

Certain of the febrile diseases have, as it were, a normal range of temperature; this has been discovered and studied especially in the malarious, typhus and typhoid fevers, small pox, scarlatina, measles, rheumatism, pyemia, pneumonia and acute tuberculosis. So characteristic is the range of temperature in each of these diseases, that it becomes a most certain means of diagnosis, although by no means the only one.

In many cases of typhoid fever which may be simulated by severe cases of pneumonia, meningitis, peritonitis, malarious fever, &c., it constitutes the only means. By it we can distinguish which stands in the causative relation in those cases called pneumo-typhoid and typho-pneumonia.

The typical range in typhoid fever is as follows:—First day morning 98.5°, evening 100.5°. 2nd day, morning 99.5°, evening 101.5°. 3d day, morning 100.5°, evening 102.5°. 4th day, morning 101.5°, evening 104°. In the second half of the first week the evening temperature is 103° to 104°, with a morning temperature one degree lower. In the last half of the first week the temperature of cases to be mild or severe so nearly coincides that it is of but little use in the prognosis; but in the second week the cases diverge so that the prognosis may almost be based upon it. In favorable cases during the second week there will be a marked difference between morning and evening temperature, even though the latter be 104° to 105°, the morning being one or two degrees lower, the range becoming less and less.

The higher the temperature the more unfavorable. A continued range of 104°, or the morning higher than the evening is a bad sign and shows danger in the third week. In the third week begin those highly characteristic daily variations, when the difference between morning and evening temperature amounts to four to six degrees, these gradually cease until convalescence is declared in the third or fourth week. In severe cases these oscillations begin earlier, the latter part of the second week. The morning temperature is as high (104° and more) and differs but little from that of the evening, or the evening succeeding, it may be even higher. These cases differ from the mild, in that the morning temperature is above the average in typhoid, which is 103° to 104° whereas in the mild it is below. Complications are apt to occur in the third week.

Therefore, if in a case presenting many of the characteristics of typhoid, we have a temperature of 103 or 104° on the first or second day, we have not a case of typhoid; likewise it holds good if on the eighth or tenth day it falls below 103°.

But time will fail me to enter into a consideration of all the diseases in which the thermometer has been especially studied, nor is it necessary for my object, which is simply to show why it should be used more extensively, and its benefits.

The literature on this subject is still in its infancy and incomplete, nor is it very accessible. I have consulted the works of Flint, Niemeyer and Aitken, especially the latter, with profit. An excellent work on this subject by Wunderlich, has just been published by the "New Sydenham Society" and is now being distributed.

The thermometer is of especial benefit in those cases where from some cause or other we can not obtain correct information from the patient, as in children, the insane, and stupidly ignorant or designing persons. We have all seen children who bear the effects of disease with wonderful fortitude, whose replies are deceptive, timidity may prevent, and extreme youth be a complete barrier to correct information from them. In all such cases the thermometer is an invaluable aid.

Many of the insane seem to be, and are, cursed, or blessed, with a complete inability to realize pain. I have seen lunatics inflict upon themselves injuries, with indifference, which a sane person could not bear without extreme suffering.

I will give a single incident from Dr. Davy which is so instructive that it will not need a single comment of mine. Dr. Davy, in making his experiments on the normal temperature of the body, found a lunatic soldier who for many weeks exhibited a temperature or 104°. His appetite was good and digestion too, so far as known. Remembering the condition to which I have alluded he examined him closely, and detected extensive disease of the lungs.

The lunatic died in a month of acute tuberculosis without any other expression of the disease than the persistant high temperature. Post mortem examination revealed ulcers of the larynx, but there had been no affection of the voice; there were vomicæ and tubercles of the lungs, but there had been no cough; there were ulcerations of the intestines, but there had been no diarrhœa; there was disease of the testes, vesiculæ seminales and also of the prostate of a severe kind, these had been equally latent during life, excepting a hardening of the testicle without pain. In this case the high temperature was the first and only indication of disease.

To the surgeon its use, it seems to me, would be of great interest and instruction in traumatic fever after wounds and operations, giving warning, at least, of those unfortunate events which render many a brilliant operation a failure.

In some instances I have been called to see female patients who had "pleurisy" and persisted in that belief; had severe pain, shortness of breath, in short they simulated pleurisy closely; but the thermometer showed no increase of temperature, and some antispasmodics cured them in a few hours. In one case the pain complained of was so severe I gave two grains of sulph. morph. with $\frac{1}{60}$ gr. sulph. atropia in less than three hours without producing any visible relief. This case was purely hysterical and within twelve hours the patient was out in the street in spite of her pleurisy and my morphia. In 1869 I was called to see a young lady suffering from an intense pain in the hip joint and extending down the thigh. Voluntary motion was extremely difficult, but drawing the limb downward or pushing it upward produced no aggravation of the pain. Two medical gentlemen had seen her, one gave her a hypodermic injection of morphia, and both were inclined to pronounce it neuralgic. There was but slight increase of the pulse and little or no fever apparent to the hand. But the thermometer showed a temperature of 101° and I gave my diagnosis as acute rheumatism, neuralgia being unaccompanied with fever, and the next day my diagnosis was confirmed.

It is of the utmost importance to keep a record of these observations, not only for future reference, but also for self defence. In regard to the latter consideration I will relate the main points of a case of importance to me, at least. A young lady aged 17 was attacked with a severe peritonitis. The first day the temperature was 100°; 2d day 102°; 3d day, morning 102½°, evening 105°; 4th day. morning 102½°, evening 103°; 5th day, morning 101¾°, evening 103¾°; 6th day, morning 101¼°, at 3 P. M. 103¼°. It will be observed that the range for the last three days closely resembles that of typhoid at a corresponding period. At this time, being in doubt as to the termination of the case, I called a medical gentleman who was on a visit to the place to see her.

She now presented a truly typhoid appearance, dusky face, slight delerium, abdominal tenderness, tympanitic, &c. My friend was so much struck with this appearance that he was inclined to pronounce it typhoid with strongly marked abdominal symptoms, although he admitted the range of temperature, which I had recorded, was against his diagnosis. But that evening the temperature sank to 102¼°, and on the seventh, or next day in the morning it was 101° and evening 102⅕°, and typhoid was excluded. The farther progress of the case substantiated my diagnosis. On the ninth day of the attack the evening temperature was 99½°. On the tenth it began to rise and on the fourteenth it stood at 104°, on the sixteenth it was down to 98½°.

During the last five days there was pain in the region of the right ovary with tenderness on pressure, the rest of the abdominal tenderness having disappeared, I regard the latter rise in temperature entirely due to the efforts of nature to establish menstruation for it was at this time just due. There was no external flow however. I mention this for I believe the menstrural epoch may and at all times will, increase the fever when occuring during the course of inflammatory affections. Now if in this case the gentleman mentioned had only seen my patient as he did and with his strong convictions of typhoid, and she had died, as there was an uncomfortable prospect of her doing, the medical millenium not being at hand, he might have said some damaging things. I was treating her boldly by the "opium plan," giving four or five grains of morphine daily, which could not, by the widest charity, be called good treatment for typhoid.

Being an older and more experienced practitioner than myself, willful or indiscreet language on his part might have seriously injured me, but I had my record by which I could have sworn it was not typhoid fever. Since suits of mal-practice are again becoming fashionable, but I am glad to say unsuccessful, I can readily conceive of the great use of such a record.

I have prepared and keep with me constantly some blanks for recording my observation at the time of making for the sake of accuracy and for comparison. Like every other instrument one must become accustomed to its use before its true value is appreciated, and when this skill is acquired it becomes not a mere instrument, but a valued guide and friend, guiding in regard to treatment and giving friendly support to diagnosis and prognosis.

When the solemn responsibilities of life and death are upon the physician, if he be wise, he will watch the conduct of that glittering, silvery little column as he would the lips of a prophet.

